# Compact Arterial Monitoring Device Use in Resuscitative Endovascular Balloon Occlusion of the Aorta (REBOA): A Simple Validation Study in Swine

**DOI:** 10.7759/cureus.70789

**Published:** 2024-10-03

**Authors:** Glen Lussier, Andrew J Evans, Isaac Houston, Andrew Wilsnack, Christopher M Russo, Robert Vietor, Peter Bedocs

**Affiliations:** 1 Department of Medicine, Uniformed Services University of the Health Sciences, Bethesda, USA; 2 Department of Anesthesiology, Walter Reed National Military Medical Center, Bethesda, USA; 3 Department of Anesthesiology, Uniformed Services University of the Health Sciences, Bethesda, USA

**Keywords:** arterial monitoring, compass transducer, hemodynamic monitoring, partial reboa, swine model

## Abstract

Introduction

Hemorrhage is the leading cause of preventable death in trauma in both the military and civilian settings worldwide. Medical studies from Operation Enduring Freedom (OEF) and Operation Iraqi Freedom (OIF) informed change in military prehospital medicine by influencing widespread tourniquet distribution and training on their use to stop life-threatening extremity hemorrhage. In the military setting, there has been a significant reduction in preventable death due to extremity exsanguination since the widespread implementation of tourniquets within the Department of Defense. However, noncompressible hemorrhage remains a significant cause of mortality, especially in the prehospital setting. In select patients, resuscitative endovascular balloon occlusion of the aorta (REBOA) is an adjunct that can be utilized to slow or stop non-compressible hemorrhage until the patient reaches definitive care.

However, frontline medical providers face the challenge of reliable, accurate blood pressure measurement in REBOA patients. REBOA, used in conjunction with a small disposable pressure monitor, can bridge the gap in capabilities, creating a more balanced resuscitation and reducing blood product requirements with the added benefit of invasive blood pressure monitoring capability.

The authors of this study propose the sustained use and further validation of a small, disposable pressure monitor in REBOA to monitor beat-to-beat variation in both hemodynamically stable and unstable patients and seek to offer a pathway for use in austere environments.

Materials and methods

Yorkshire swine (n = 4) were selected for partial REBOA (pREBOA) placement and compass transducer measurement in conjunction with a vascular experimental protocol. Appropriate vascular and arterial line access was obtained, hemorrhagic shock was initiated, and REBOA with an in-line Compass™ device (CD) pressure transducer (Centurion Medical Products, Williamston, MI) was used to occlude the aorta. Mean arterial pressures were measured via the CD, recorded, and compared to the control arterial line at hypotensive, normotensive, and hypertensive pressures.

Results

At hypotensive pressures, 30% of the CD readings fell within 1 mmHg of control arterial line readings, and 52.3% were within 2 mmHg. At normotensive pressures, 46% of the CD readings fell within 1 mmHg of control arterial line readings, and 64.2% were within 2 mmHg. At hypertensive pressures, 60% of the CD readings fell within 1 mmHg of control arterial line readings, and 82% were within 2 mmHg. All CD data points at all pressures were within 8 mmHg of the control arterial line readings.

Conclusions

In conclusion, the CD is a compact, inexpensive, portable pressure-sensing device that may potentially augment the safety and functionality of the REBOA in trauma patients both at the point of injury and in the hospital. This novel study conducted on four swine subjects demonstrated a remarkable correlation to the traditional equipment intensive arterial line setups, and issues of stasis and non-pulsatility were easily troubleshot. Future studies should investigate CD use in REBOA catheters under different physiological conditions, specifically arrhythmias, and in different environments (prehospital, air medical transport, and austere locations).

## Introduction

Hemorrhage is the leading cause of preventable death in trauma in both the military and civilian settings worldwide. Medical studies from Operation Enduring Freedom (OEF) and Operation Iraqi Freedom (OIF) informed change in military prehospital medicine by influencing widespread tourniquet distribution and training on their use to stop life-threatening extremity hemorrhage in the setting of trauma. In the military setting, there has been an 85% reduction in preventable death due to extremity exsanguination since the widespread implementation of tourniquets within the Department of Defense [1]. However, noncompressible hemorrhage remains a significant cause of mortality, especially in the prehospital setting. The most common sites of lethal injury from non-compressible hemorrhage are from the chest and abdomen (67.3%), junctional (19.2%), and peripheral extremity (13.5%). In select patients, resuscitative endovascular balloon occlusion of the aorta (REBOA) is an adjunct that can be utilized to slow or stop non-compressible hemorrhage until the patient reaches definitive care [1].

REBOA has shown a survival benefit in patients experiencing non-compressible truncal hemorrhage. REBOA has been tested and approved for various traumatic and surgical pathology, including abdominal and/or pelvic trauma, perioperative surgical bleeding, post-partum hemorrhage (PPH), and aortic rupture and is now considered a mainstay treatment for endovascular hemorrhage control by various training medical evacuation platforms, namely, the American College of Surgeons Basic Endovascular Skills for Trauma (BEST) and Endovascular Skills for Trauma and Resuscitative Surgery (ESTARS) courses. Increased utilization and continual technical improvements of both the device/equipment and deployment techniques have resulted in REBOA being successfully utilized in austere operational settings and military medicine [2-5]. In addition to stopping and slowing hemorrhage, REBOA can also be used to measure arterial blood pressure in real-time with an arterial pressure measuring device to guide volume resuscitation until arrival to a facility with trauma surgical and intensive care capabilities. In both the operational military and austere environments, logistics and transport capabilities are often the limiting factors for trauma patients to receive the care they need. Space and supplies are, by nature, limited in these settings to what a provider can carry in a backpack, vehicle, or makeshift clinic. Early targeted volume resuscitation in the trauma patient is preferred, with multicomponent blood products or whole blood when available. When providing trauma resuscitation in resource-poor settings without the luxuries of the tertiary care medical centers in the United States, seemingly simple resources, such as blood products, medications, and functional emergency equipment, save lives. Aside from transporting various product coolers, which increases both the footprint and manpower to transport, blood products are a perishable, unstable resource requiring temperature-regulated storage. Identifying this complex, multidisciplinary, essential issue, the United States Army Rangers developed the “walking blood bank.” Ranger O Low Titer (ROLO) forward-walking blood bank utilizes pre-identified donors and ensures fast, reliable, and fresh blood products while not increasing the logistical burden to aid in trauma resuscitation [6]. This unique practice requires much pre-deployment testing to ensure deploying service members have accurate blood types and are without any transferable blood-borne infectious diseases. Once all the service members of the deploying unit have given their blood samples, the members with group O blood type are separated, and an additional blood sample is collected to determine their individual titer levels. Low titer levels are associated with a significantly lower likelihood of acute transfusion reactions and are preferentially selected by the program manager. This tedious process results in critical pre-deployment information and identification of what service members have universal donors (whose blood can be transfused into anyone).

With more reliable and accessible access to blood products in the deployed setting, we face the challenge of reliable, accurate, and dynamic blood pressure measurement. Traditional arterial line setups are bulky and cumbersome and require advanced monitoring devices. REBOA, used in conjunction with a small disposable pressure monitor, can bridge the gap in capabilities, creating a more balanced resuscitation and reducing blood product requirements with the added benefit of invasive blood pressure monitoring capability [7-9]. This small disposal pressure monitor, the Centurion Compass Universal Hg Device (CCUHD) (Centurion Medical Products, Williamston, MI), is described below.

The Compass™ device (CD) (Centurion Medical Products, Williamston, MI) is a compact self-calibrating device with a liquid crystal display (LCD) screen approved by the Food and Drug Administration (FDA). The CD can be used to measure physiologic pressure and has been validated for use in the placement of central venous catheters, monitoring of central venous pressures, extremity compartment pressures, intrathoracic pressures, intra-abdominal pressures, and intrathecal pressure [10]. Recently, the CD has been used in tandem with REBOA to help mitigate its limitations in the austere environment.

The REBOA/CD setup has undergone feasibility studies in prehospital medical cardiac arrest patients to determine mean arterial pressures (MAPs) to guide cardiopulmonary resuscitation [11]. Schmid et al. [11] compared MAP measurements from the CD to the standard arterial line setup (transducer and pressure bag setup) on hemodynamically stable critical care patients [12]. Schmid et al. found that the CD provided a close estimate for MAP. The CD was shown to provide lower pressure measurements when MAPs were lower, and higher estimates with MAPs were higher using the current standard of care arterial line measurement method of transducer and pressure bag [13]. The REBOA/CD has also been used to study aid in the resuscitation of traumatically wounded hypovolemic patients. In 2021, Holtestaul et al. [14] tested and assessed the feasibility of maintaining permissive hypotension during intermittent REBOA in a porcine model guided by CD monitoring [14]. They tested the ability of the CD to guide REBOA placement and titration in patients in hypovolemic shock and validated the CD for use in an austere setting where traditional arterial monitoring was unavailable. They concluded that the CD strongly correlates with traditional arterial line measurements, showing an average difference of 6 mmHg in SBPs measured 8-196 mmHg and an average of 3 mmHg in MAPs <65 mmHg. They also validated using the CD to aid in resuscitation using an intermittent inflation protocol to maintain MAP >40 mmHg. High accuracy at hypotensive pressures and can be used to guide intermittent REBOA.

Benham et al. [15] evaluated the accuracy and precision of the CD compared to standard pressure monitoring using REBOA in uninjured and injured patient sets [15]. The results showed a strong correlation in the accuracy and precision of proximal and distal CD compared to traditional arterial pressure monitoring of uninjured and injured patients. The injured patient set showed a low bias for the CD and traditional arterial line measurements in both the proximal and distal pressure measurements. They concluded that the CD/REBOA combination could be used as an alternative to traditional arterial blood pressure monitoring in a resource-limited or deployed environment.

The authors of this study propose the sustained use of the CD in REBOA to monitor beat-to-beat variation in both hemodynamically stable and unstable patients. The authors seek to offer further validation studies of CD use in REBOA and provide a pathway for use in austere environments.

## Materials and methods

Under a Uniformed Services University Institutional Animal Care and Use Committee (IUCAC) protocol (SUR-19-965; assurance number D-16-00285), four Yorkshire swine (45-57 kg) were selected for partial REBOA (pREBOA) placement and compass transducer measurement in conjunction with a vascular experimental protocol conducted simultaneously. All experiments were conducted in accordance with protocol SUR-19-965 and approved by the Designated Member Review committee. This sample size was chosen to limit live-tissue experimentation while still providing sufficient data to meet experimental objectives. All swine were cared for under guidelines published by the Institute of Laboratory Animal Research's Guide for the Care and Use of Laboratory Animals.

To initiate the experiment, Yorkshire swine were premedicated with ketamine intramuscularly. Following isoflurane induction and endotracheal intubation, maintenance anesthesia was achieved with isoflurane. To offset the vasodilatory effects of general anesthesia, an intravenous infusion of norepinephrine (0.01 μg/kg per hour) was instituted upon venous access and titrated to achieve a target MAP between 65 and 75 mmHg. The infusion rate remained constant for the remainder of the experiment. Animals were mechanically ventilated with tidal volumes of 7 to 10 mL/kg and a respiratory rate of 10 to 15 breaths per minute sufficient to maintain normocapnia. Isotonic sodium chloride solution was administered at a rate of 5 mL/kg per hour to overcome insensible losses. An underbody warmer set at 39°C was used to maintain body temperature.

For vascular access, a 5 Fr microcatheter was placed into the brachial artery for arterial hemodynamic monitoring using a modified Seldinger technique. An appropriate arterial waveform was confirmed on the transducer and was labeled as the brachial arterial line (BAL) in LabChart (ADInstruments, Dunedin, New Zealand). Central venous access was achieved in the right internal jugular vein with percutaneous access. Given the duration of the experiment, a Foley catheter was placed through open surgical cystotomy.

After appropriate vascular and arterial line access was obtained, hemorrhagic shock was initiated by the surgical team with traumatic amputation of the liver (approximately 30% total liver volume) followed by 90 seconds of free hemorrhage. The REBOA was then placed through right femoral artery access. Complete occlusion of the aorta was achieved with REBOA 1.5 minutes following the initiation of injury. Following 20 minutes of zone 1 aortic occlusion, permissive regional hypoperfusion with partial aortic occlusion (pREBOA) occurred. The study animals underwent damage control surgery with definitive hemorrhage control and resuscitation with whole blood. Blood volume was returned through a central venous line via rapid infusion at the rate of 150-1,000 cc/minute, which allowed for hypotensive, normotensive, and hypertensive MAPs to be obtained while the REBOA was in place.

**Figure 1:**
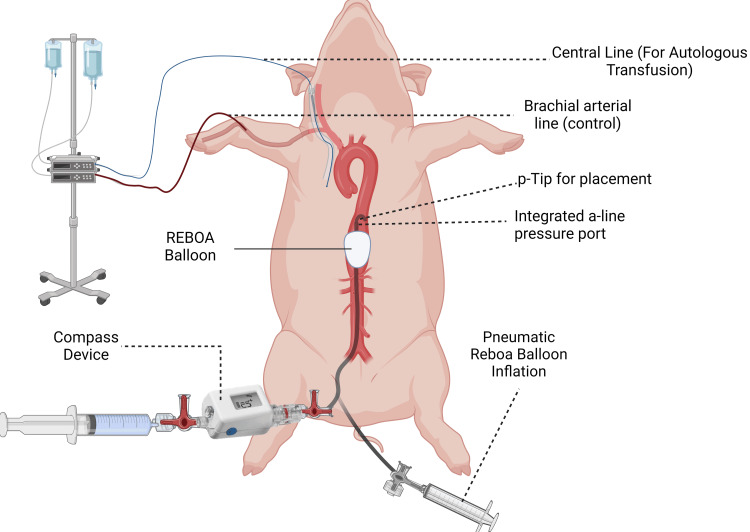
Diagram of experimental design with invasive line placement This figure is the original work of the authors via BioRender (BioRender Inc., Toronto, ON, Canada) REBOA, resuscitative endovascular balloon occlusion of the aorta

After REBOA placement, the CD was attached to the proximal port of the REBOA catheter, and MAP was noted by experimenters. An illustration is provided in Figure 1. Vital signs, brachial arterial pressures, and CD measurements were recorded by the experimenters with assistance from veterinary technicians.

Once target MAPs were achieved on the BAL transducer, pulsatile flow was confirmed on the CD. One experimenter was blinded to the BAL and the other to the CD. At a predetermined time interval of 10 seconds, the MAP displayed on the CD was announced and recorded into LabChart. Data were continually recorded for 10 data points, and a 180-second interlude was conducted without data collection. Data were then collected again for at least 100 data points per swine at the target MAP. If non-pulsatile flow was detected on the CD, the CD was flushed with 10 mL of NS at the proximal port. After flushing, pulsatile flow returned, and experimental methods were continued according to the above procedures.

At the conclusion of the vascular protocol and CD experiment, the REBOA catheter was removed, and the study animals completed a 240-minute critical care study. Euthanasia was conducted in accordance with the most current American Veterinary Medicine Association (AVMA) Guidelines for the Euthanasia of Animals. A pentobarbital-based euthanasia solution affected euthanasia (Euthasol, 100 mg/kg, 1 mL/10 pounds body weight) while still under general anesthesia. Death was confirmed with blood pressure and heart rate evaluation.

CD correlation with BAL was assessed using a Bland-Altman plot to determine agreement between data points. Systematic biases were identified with the plot to determine any skew from the average between the two measurements.

## Results

**Figure 2:**
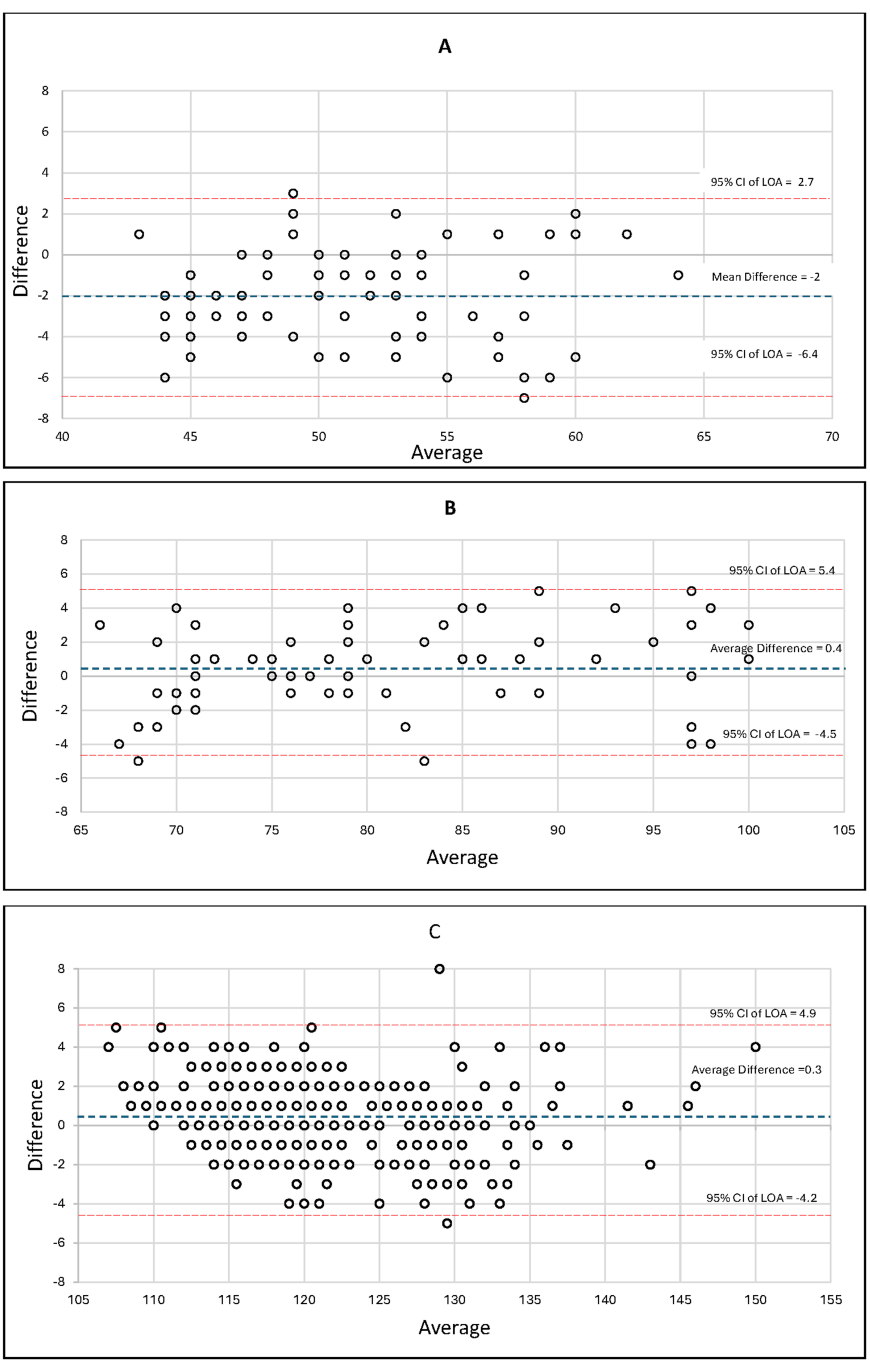
Bland-Altom plot displaying hypotensive, normotensive, and hypertensive MAPs A: average versus difference MAP 40-64; B: average versus difference MAP 65-101; C: average versus difference MAP 102-150 LoA: limits of agreement; MAP, mean arterial pressure

The following Bland-Altman plots demonstrate CD correlation with the control BAL measurements, as seen in Figure 2. For hypotensive MAPs (n = 27), 30% of the compass readings fell between 1 mmHg, while normotensive (n = 31) and hypertensive MAPs (n = 266) fell within 46% and 60%, respectively. These data improve within 2 mmHg of the BAL to 52.3%, 64.2%, and 82% for hypotensive (n = 46), normotensive (n = 43), and hypertensive (n = 363) MAPs. All data points (n = 606) were within 8 mmHg MAPs unless there was evidence of non-pulsatile flow, in which the proximal port was flushed according to the experimental protocol.

Statistical analysis

**Figure 3:**
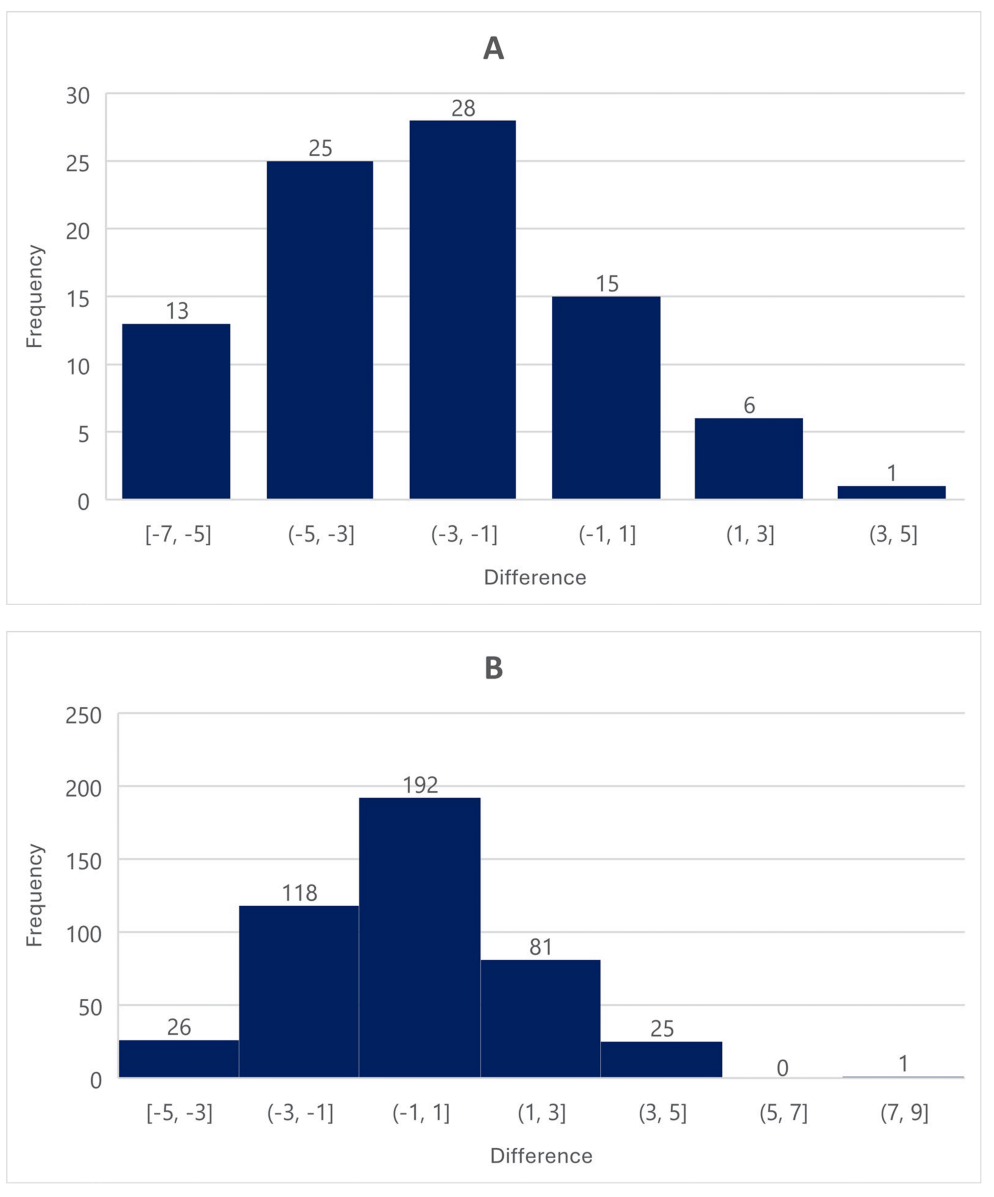
Frequency of difference of MAPs for hypotensive and hypertensive data sets A: frequency of difference MAP 40-64; B: frequency of difference MAP 102-150 MAP: mean arterial pressure

As demonstrated in Figure 2 (Bland-Altom plot displaying hypotensive, normotensive, and hypertensive MAPs) and Figure 3 (frequency of MAP differences), CD readings resemble a distribution curve with evidence of systemic bias in all groups. The average difference from the BAL for hypotensive, normotensive, and hypertensive MAPs were -2, 0.4, and 0.3, respectively. The standard deviations for the hypotensive (n = 88), normotensive (n = 67), and hypertensive (n = 443) MAPs were 2.41, 2.51, and 1.88, respectively. The limits of agreement at the 95% confidence interval were -6.7 (lower limit) and 2.7 (upper limit) for hypotensive MAPs, -4.5 (lower limit) and 5.4 (upper limit) for normotensive MAPs, and -4.2 (lower limit) and 4.9 (upper limit) for hypertensive MAPs as expressed in Figure 2. The average difference for hypotensive CD MAP readings trended lower with the new method compared to the BAL, while normotensive and hypertensive CD MAP readings trended slightly higher, as demonstrated in Figure 3. In other words, the systemic bias of the new method was dependent on the MAP range, as hypotensive MAPs were consistently negative, while normotensive and hypertensive MAPs were consistently positive.

Anomalies and non-pulsatile flow were also documented according to the protocol mentioned above. Anomalies were defined as readings of less than or greater than 10 mmHg variation between the CD and the traditional arterial line setup. This occurred for seven of the 606 total data points obtained (1.1%). As data were recorded, recognition between investigators was made if the CD MAP was anomalous. Then, the pulsatile flow was checked to determine the internal validity of the CD reading. With the blinded comparison of CD to MAPs, every instance of an anomalous CD MAP correlated with poor pulsatile flow. Following the identification of poor pulsatile flow, the CD was flushed with 10 mL of sterile saline. Following initiation of the full flush, the CD began reading with a MAP correlate of +5/-5 within 30 seconds (three measurements) of return to pulsatility in every instance. This primarily occurred at hypotensive or normotensive MAP readings, with no loss of pulsatile flow with arterial line MAPs >101.

## Discussion

Data obtained from the four live tissue trials demonstrate agreeability in BAL and CD in hypotensive, normotensive, and hypertensive swine models; 95% of all data points conservatively fell between 7 mmHg of the control across multiple pressure ranges. This in vivo swine study supported earlier work, giving credence to the use of CDs in the placement of REBOA catheters to monitor MAPs. The CD, in conjunction with the REBOA, showed a high positive correlation of MAPs when compared to the traditional equipment-intensive arterial line transducer. It is the experimenter’s opinion that this further adds to the validity of the CD and may one day be used in pre-hospital or austere environments as a surrogate to the traditional arterial line transducer in REBOA patients. However, further human validation studies in such environments are necessary. 

Troubleshooting the CD

An important component of this study is establishing its translation to the austere and prehospital environment. In hemodynamically unstable or hypercoagulable patients, the CD may lose pulsatile flow. Data collected here suggest that flushing of the line (presumed clot formation at the transducer tip) may be an appropriate remedy for non-pulsatile flow. However, the clinician would need to consider other potential causes of non-pulsatile flow to include transducer misplacement against the wall of the vasculature, transport of the patient affecting the transducer, proximal obstruction (REBOA occlusion), patient factors (gagging, coughing), and intra-abdominal pressure shifts (abdominal compartment syndromes). Regardless, the non-pulsatile flow indication is a beneficial data point for informing clinical decisions in austere environments, and flushing the line with saline may be an appropriate intervention.

Limitations

Limitations of this study were the sample size (n = 4), duration of the study (90 minutes), study location (clinical/laboratory environment), and blinding. Previous validation studies for the CD ranged from a sample size of n = 4-40, used uninjured and injured hemodynamically stable and unstable subjects, and had a duration from 10-120 minutes (upper threshold for REBOA inflation) [12,14,15]. Other studies may benefit from longer-duration experiments, especially to validate in a prolonged field care-type scenario. Per the manufacturer, the CD has a battery life of eight hours. For longer-term use outside of two hours, future studies could determine if the precision/accuracy/reliability of the CD is affected by battery life. In terms of study location, future studies could test the CD for use as a stand-alone arterial pressure monitoring device in an austere setting for conditions other than traumatic hemorrhage (arrhythmia or CPR), which may also necessitate invasive arterial monitoring capability.

Finally, the study above only blinded the researchers to a single value. One researcher was blinded to the BAL, and the other was blinded to the CD. Measurements were called out to be recorded in LabChart, meaning both researchers at the time of the experiment were immediately privy to the agreeability of the data. Conducting a further blinded study in the future could mitigate any bias from the researchers in data collection. For example, researchers could chart data separately at predetermined time intervals on synchronized time-keeping devices. Despite the aforementioned limitations, the data above give credence to CD use in prehospital environments.

## Conclusions

In conclusion, the CD is a compact, precise, and expedient pressure-sensing device that augments the safety and functionality of the REBOA in trauma patients in and out of the hospital. The CD/REBOA system demonstrated a remarkable correlation to the traditional equipment-intensive BAL setups, and issues of stasis and non-pulsatility were easily troubleshooted. Future studies should investigate CD use in REBOA catheters under different physiological conditions, specifically arrhythmias, and in different environments (prehospital, air medical transport, and austere locations).
